# The HIV care cascade: models, measures and moving forward

**DOI:** 10.7448/IAS.18.1.19395

**Published:** 2015-03-02

**Authors:** Sarah MacCarthy, Michael Hoffmann, Laura Ferguson, Amy Nunn, Risha Irvin, David Bangsberg, Sofia Gruskin, Ines Dourado

**Affiliations:** 1 RAND Corporation, Santa Monica, CA, USA; 2 Division of Infectious Diseases, The Miriam Hospital, Alpert Medical School of Brown University, Providence, RI, USA; 3 Program on Global Health and Human Rights, Institute for Global Health, University of Southern California, Los Angeles, CA, USA; 4 Division of Infectious Diseases, Johns Hopkins University, Baltimore, MD, USA; 5 Center for Global Health, Massachusetts General Hospital, Boston, MA, USA; 6 Ragon Institute of Massachusetts General Hospital, Massachusetts Institute of Technology, Harvard Medical School, Cambridge, MA, USA; 7 Institute of Collective Health, Federal University of Bahia, Salvador, Brazil

**Keywords:** HIV/AIDS, care cascade, treatment cascade, continuum of care, testing, linkage to care, viral suppression, measures

## Abstract

**Introduction:**

This article seeks to identify where delays occur along the adult HIV care cascade (“the cascade”), to improve understanding of what constitutes “delay” at each stage of the cascade and how this can be measured across a range of settings and to inform service delivery efforts. Current metrics are reviewed, measures informed by global guidelines are suggested and areas for further clarification are underscored.

**Discussion:**

Questions remain on how best to evaluate late entry into each stage of the cascade. The delayed uptake of HIV testing may be more consistently measured once rapid CD4 testing is administered at the time of HIV testing. For late enrolment, preliminary research has begun to determine how different time intervals for linking to HIV care affect individual health. Regarding treatment, since 2013, the World Health Organization (WHO) and UNAIDS recommend treatment initiation when CD4 <500 cells/mm^3^; these guidelines provide a useful albeit evolving threshold to define late treatment initiation. Finally, WHO guidelines for high-, low- and middle-income countries also could be used to standardize measures for achieving viral suppression.

**Conclusions:**

There is no “one size fits all” model as the provision of services may differ based on a range of factors. Nonetheless, measures informed by global guidelines are needed to more consistently evaluate the scope of and factors associated with delays to each stage of the cascade. Doing so will help identify how practitioners can best deliver services and facilitate access to and continued engagement in care.

## Introduction

The advent of antiretroviral therapy (ART) was meant to change the course of the AIDS epidemic. In many ways it did: there was a dramatic reduction in AIDS-related morbidity and mortality, and in some contexts HIV/AIDS is now a manageable chronic illness. Yet, in high- and low-income countries alike, studies continue to document delays in the utilization of available services, including HIV counselling and testing, treatment and continued engagement in care services. These delays are correlated with compromised treatment response and missed opportunities for preventing HIV transmission [1]. The HPTN 052 study published by Cohen and colleagues demonstrated a 96% reduction in the number of linked HIV-1 transmissions due to early treatment initiation [2]. These results further illustrate the importance of earlier ART initiation to achieve viral suppression, the mechanism highlighted by authors as largely responsible for decreased transmission rates. Notably, few HIV-positive individuals reach viral suppression. In 2012, for example, the US Centers for Disease Control and Prevention (CDC) released data indicating that in the United States, only 25% of individuals living with HIV were sufficiently engaged in care to achieve viral suppression [3]. As such, substantial evidence suggests that both access to services and the timing in which services are reached are critically important to improving individual and population health outcomes.

But it remains unclear how best to measure the timing in which services are accessed, further complicating efforts to identify the factors associated with delayed presentation to particular stages of the adult HIV care cascade (hereafter referred to as the cascade). Furthermore, recent reviews from both high-income [4,5] and low-income [6,7] contexts have highlighted definitional questions regarding the cascade and underscore the need for greater clarity. A closer analysis of different models and associated measures of the cascade across settings remains lacking.

Therefore, we review the differences between models of the cascade and discuss how the focus on retention within each stage of care does not evaluate late entry into care. Drawing from global guidelines produced by the World Health Organization (WHO), suggestions are made regarding how to evaluate late entry into each stage of the cascade across settings. Identifying the variety of measures used can critically inform data analysis for cross-country comparisons and the improvement of service provision across a range of settings.

## Discussion

### Models of the care cascade

Current models of the cascade differ in how they outline the stages of HIV services needed for an HIV-positive individual to achieve viral suppression (Table 1). The US CDC model [3,8,9] identifies five primary stages including HIV testing and diagnosis, linkage to care, retention in care, treatment and viral suppression. Likewise, the British HIV Association outlines similar steps in its Standard of Care Guidelines and includes a range of measurable outcomes for both attrition and timely engagement in HIV care across a range of HIV services [10]. Comparable versions of the cascade are being used in other country contexts as well. The Brazilian Ministry of Health, for example, recently adopted a model of the cascade similar to the CDC version, adapting its measurements to the availability of national data [11]. Shifting to the global level, in 2013, WHO provided a more detailed model of the cascade, identifying several different sub-stages [12]. In sum, there are various models of the cascade; however, common elements of the cascade include testing, linkage or enrolment, CD4 testing, retention in care, treatment initiation and viral suppression.

**Table 1:** Models and measures of the adult care cascade outlining the stages of HIV services needed for an HIV-positive individual to achieve viral suppression

Model	Testing/diagnosis	Linkage/enrolment	Retained in care	Treatment	Viral suppression
US [3,8,9]	Number of HIV-diagnosed cases divided by estimated number of infected cases.	Estimated number of PLWHA with ≥1 CD4 or VL within a 12-month period divided by estimated number of infected cases.	Estimated number of PLWHA with ≥2 CD4 or VL 3 months apart with a 12-month period divided by estimated number of infected cases.	Estimated number of PLWHA on ART divided by estimated number of infected cases.	Estimated number of PLWHA with undetectable viral load divided by estimated number infected of cases.
UK [10]^a^	An HIV-positive diagnosis with a CD4 below 350. Very late is below 200.	Initial meeting with a specialist should be no later than 2 weeks after receiving a positive test result, which should be delivered to the person within 48 hours.	Proportion of people newly diagnosed with HIV who have a CD4 count result in their clinical record within 1 month of their HIV diagnosis (target: >95%).	Proportion of new patients who start therapy when indicated with a CD4 count of <350 cells/mm^3^ while not already on therapy.	Patients with HIV viral load assessed within 6 weeks of commencing ART (target: 95%).
Brazil [11]	Number of HIV-diagnosed cases.	Number of PLWHA who have been linked to health services and have had CD4 and viral load counts or are on ART treatment.	Number of PLWHA that have continued laboratory monitoring or ART therapy throughout the period analyzed.	Number of PLWHA on ART.	Number of PLWHA presenting undetectable viral load (<=50 copies/mL).
WHO [12]^b^	HIV testing and counselling.	Linkage to care serves as an intermediary step to reach the next stage of enrolment in care.	Retained in care: HIV prevention, HIV care, ART preparation, managing co-infections and comorbidities is the intermediary step between enrolment in care and first line ART.	ART initiation (first, second, third line ART). Late treatment: those initiating ART with CD4 less ≤500 cells/mm^3^, except under special circumstances.	Viral suppression is achieved when an individual has <1000 RNA copies/mL in low and middle-income countries and <50 RNA copies/mL in high-income countries [13].

a The BHIV guidelines include several measurable outcomes for evaluating attrition and delay across certain stages of care. We include an example of measurable outcome for each stage as indicated by the BHIV guidelines;

b The WHO does not offer explicit parameters or timeframes for successful entry into each stage of care.

There are several critical differences between models that are worth highlighting. First, they primarily evaluate retention within each stage, for example, the number or percentage of HIV-positive individuals successfully entering each stage of care. Although important, this is distinct from measuring the number or percentage of individuals presenting late to an HIV service. In terms of models, the CDC cascade includes an element of time in its measures for enrolment and retention in care, whereas the WHO does not highlight time at all. This leads to a more general point regarding the cascade: determining the appropriate threshold by which “late” can be measured is challenging, especially in the absence of clinical guidelines. Furthermore, even when clinical guidelines exist, different thresholds are commonly used, making it difficult to determine a standard across different contexts by which late access to each stage of the cascade could be measured.

It is important to note that there is no “one size fits all” model of the cascade as the provision of services may differ based on a range of factors such as the country context and population of interest. For example, the standard for viral suppression or treatment initiation varies among high-income countries compared to low- and middle-income countries [13]. With respect to the population of interest, the flow of services may also differ based on specific characteristics. Pregnant women and sero-discordant couples, for example, may be prioritized differently due to risk of HIV transmission. Even within these populations, the point of entry into care, especially as it shifts from facility-based testing to home-based testing in some contexts, may further influence patients’ care trajectory. It remains critical to acknowledge the economic, political and social contexts surrounding each epidemic. Therefore, country-specific measures that take national guidelines into account may also be useful. However, to increase universal comparability across contexts, where possible, measures informed by global guidelines could be helpful, recognizing that differences will likely persist given the common disconnect between national and global guidelines.

In this article, we highlight how existing global guidelines could inform efforts to evaluate late entry into each stage of the cascade. Where global guidelines are lacking, we highlight what areas of additional information are needed. Importantly, we use global guidelines to inform this analysis given that WHO has a systematic process by which a network of diverse experts analyze evidence aggregated by the GRADE methodology to ensure guidelines are developed through a transparent, evidence-based process [14].

### Measures for the care cascade

Recognizing that diverse approaches may be required, this section seeks to review how delays at each stage are commonly measured (Table 2), and when global guidelines permit, suggest measures to improve the comparability of delayed entry to different stages of the cascade across contexts.

**Table 2:** Measures of delay for the adult care cascade employed in the peer-reviewed literature

	Testing/diagnosis	Linkage/enrolment	Retained in care	Treatment	Viral suppression
Peer-reviewed literature	Late testing is commonly defined in the peer-reviewed literature in terms of concurrent diagnoses of HIV and AIDS [15–19] or as a measurement of time between HIV and AIDS diagnosis [20–24].	Late enrolment is defined in relation to the time between HIV diagnosis and presentation to a wide range of HIV-related services including enrolment, CD4 evaluation and treatment initiation.	Different retention measures and their association with the likelihood of individuals achieving viral suppression have been employed [25–28].	Late treatment is measured multiple ways. WHO and UNAIDS recommend treatment initiation when CD4 <500 cells/mm^3^ or with an AIDS-defining event, regardless of CD4 count at the time of treatment initiation.	A majority of studies used thresholds in the 300–500 RNA copies/mL range [13].

HIV testing is generally measured by the percentage of people who are tested for HIV in a given population. Late testing is commonly defined in the peer-reviewed literature in terms of concurrent diagnoses of HIV and AIDS [15–19] or as a measurement of time between HIV and AIDS diagnosis [20–24]. Although measures defining late testing in relation to AIDS diagnosis on average would reflect late testing, fast and slow progressors exist, which may limit the effectiveness of these definitions. Despite this potential limitation, these measures appear to be the best proxy measure of late testing at this time. An ideal measure would evaluate the time from HIV infection to diagnosis. The WHO has established a technical working group to develop an incidence assay that would define recent infection at the time of testing; however, such technology is currently only available at a population level and cannot estimate individual infection time [29]. Point-of-care CD4 testing could also provide a practical alternative to more effectively measure late testing. To date, however, this technology is not widely available, and even once it is accessible, the differences in disease progression will continue to affect the ability to clearly and consistently define “late” testers.

Linkage or enrolment is commonly evaluated as the percentage of people who have been diagnosed with HIV that enrolled in care. Late enrolment is defined in relation to the time between HIV diagnosis and presentation to a wide range of HIV-related services, including enrolment, CD4 evaluation and treatment initiation. Recent studies have begun to quantify the relationship between different retention measures and their association with the likelihood of individuals achieving viral suppression [25–28]. Although these findings may provide useful insights for the evaluation of late enrolment, global guidelines have yet to make clear statements regarding important thresholds by which a patient should enrol in care. Furthermore, differences in service provision (for example, CD4 evaluation prior to or following enrolment in HIV-related services) as well as differences in the package of what constitutes care (such as treatment regardless of CD4 vs. treatment initiation only once CD4 drops below a specific threshold) can further affect how late enrolment is determined.

As ART has become more widely available, the percentage of people on ART has been used as the catchall measure reflecting successful engagement in care. Late treatment initiation has been measured in several different ways. As of 2013, WHO and UNAIDS recommend treatment initiation when CD4 <500 cells/mm^3^ or with an AIDS-defining event, regardless of CD4 count at the time of treatment initiation. However, as research on HIV treatment continues to suggest that earlier initiation of treatment can prevent disease progression, limit damage to organ systems and minimize the risk of transmission, CD4 cell count may play less of a role in the decision to initiate treatment, particularly in high-income settings.

Viral load suppression, the desired outcome of timely advancement along the cascade, is also inconsistently defined. According to WHO guidelines, viral suppression is achieved when an individual has <1000 RNA copies/mL in low- and middle-income countries and <50 RNA copies/mL in high-income countries [13]. However, a recent literature review on low- and middle-income countries [13] reported that the majority of studies used thresholds in the 300–500 RNA copies/mL range. At this time, WHO guidelines for high-, low-, and middle-income countries could be used to standardize measures for achieving viral suppression. By using a range of cost-effective techniques, point-of-care viral load testing could be expanded in limited resource settings and offer opportunities to standardize measures [30]. Specifically, expanding viral load testing may help determine if distinct viral RNA thresholds across economic contexts are appropriate.

## Conclusions

Our review of several models and their associated measures highlights major differences and areas for further clarification within the cascade. There appears to be consensus around how CD4 count can help define “late” but more work is needed so that global guidelines can better inform future measures. Point-of-care CD4 count tests could substantially improve our ability to measure what constitutes late presentation to a range of services, though as evidence mounts in support of earlier treatment initiation, the importance of CD4 evaluations may be further diminished [31]. WHO now recommends viral load testing as the preferred approach to monitoring ART success and diagnosing treatment failure; thus, if access to viral load testing does in fact increase [30], it might become a more useful measure for assessing delays in uptake of HIV testing and treatment initiation as well as determining when viral suppression is achieved.

Although this article focuses on differences between global and national guidelines, there are also differences at the local level to be discussed. For example, some health departments have developed best practices, including same day appointments along with transportation and peer support when needed, which has been shown to increase the likelihood of enrolment [32]. In other settings, all people living with HIV attending HIV care services are provided co-trimoxazole prophylaxis, which may serve as a motivator for people who are not yet ART-eligible to remain engaged in care [33]. As such, consideration of locally implemented guidelines may also help identify strategies that promote timely access and continued engagement in care.

Our final points perhaps lead to the next step in considering how the cascade is conceptualized more broadly. Currently, models are presented as linear such that a patient, once testing positive, ideally transitions to the next stage of care. However, stages along the cascade must be repeated: HIV testing (for those who test negative) requires HIV-negative people to stay engaged in care; CD4 count and viral load testing (for those who test positive) should be repeated regularly. In addition, many patients cycle in and out of care over the life course [34]. Re-presentation to care after initial loss to follow up is one of the most challenging aspects of the cascade to measure, and future work should seek to develop retention metrics that can be adapted to capture this complexity across settings. Furthermore, efforts to situate the cascade within the larger context of primary care may reinforce attempts to routinize HIV counselling and testing and to ensure that the comprehensive health needs of HIV-positive patients are met. Ultimately, a closer look at the differences among models and measures of the cascade will help identify how practitioners can best deliver services. Global guidelines in particular can better inform how more consistent measures of “delay” are used across settings and in turn improve both individual and population health. Only in so doing do we have a chance to not only change the course of this epidemic but to halt it.
